# Potent antitumor activity of a glutamyltransferase-derived peptide via an activation of oncosis pathway

**DOI:** 10.1038/s41598-021-93055-5

**Published:** 2021-08-13

**Authors:** Cheng Fang, Wenhui Li, Ruozhe Yin, Donglie Zhu, Xing Liu, Huihui Wu, Qingqiang Wang, Wenwen Wang, Quan Bai, Biliang Chen, Xuebiao Yao, Yong Chen

**Affiliations:** 1 Department of Hepatic Surgery IV, Third Affiliated Hospital of Second Military Medical University, Shanghai, 200438 China; 2 Department of Gynecology and Obstetrics, First Affiliated Hospital of Second Military Medical University, Shanghai, 200438 China; 3 Department of Hepatobiliary Surgery, Xijing Hospital, Fourth Military Medical University, Xi’an, 710032 China; 4 Anhui Key Laboratory for Cellular Dynamics and Chemical Biology, Hefei National Science Center, University of Science and Technology of China, Hefei, 230000 China; 5 Key Lab of Modern Separation Science in Shaanxi Province, Northwest University, Xi’an 710069, China; 6 Department of Gynecology and Obstetrics, Xijing Hospital, Fourth Military Medical University, Xi’an, 710032 China

**Keywords:** Drug discovery, Medical research

## Abstract

Hepatocellular carcinoma (HCC) still presents poor prognosis with high mortality rate, despite of the improvement in the management. The challenge for precision treatment was due to the fact that little targeted therapeutics are available for HCC. Recent studies show that metabolic and circulating peptides serve as endogenous switches for correcting aberrant cellular plasticity. Here we explored the antitumor activity of low molecular components in human umbilical serum and identified a high abundance peptide VI-13 by peptidome analysis, which was recognized as the part of glutamyltransferase signal peptide. We modified VI-13 by inserting four arginines and obtained an analog peptide VI-17 to improve its solubility. Our analyses showed that the peptide VI-17 induced rapid context-dependent cell death, and exhibited a higher sensitivity on hepatoma cells, which is attenuated by polyethylene glycol but not necrotic inhibitors such as z-VAD-fmk or necrostatin-1. Morphologically, VI-17 induced cell swelling, blebbing and membrane rupture with release of cellular ATP and LDH into extracellular media, which is hallmark of oncotic process. Mechanistically, VI-17 induced cell membrane pore formation, degradation of α-tubulin via influx of calcium ion. These results indicated that the novel peptide VI-17 induced oncosis in HCC cells, which could serve as a promising lead for development of therapeutic intervention of HCC.

## Introduction

Hepatocellular carcinoma (HCC) presents a major health threat in South-East Asia, especially in China. It ranks third among all malignancies both in incidence and mortality in China and accounts for approximately 42.5% of the total incidence worldwide despite the improved surveillance for high-risk patients and surgical intervention^1,2^. HCC remains challenging to treat, owing to a paucity of drugs that target critical and context dependencies^3,4^; broad-spectrum kinase inhibitors such as sorafenib provide only a modest benefit to patients with HCC^5^. Several lines of recent studies exhibit promising potential in exploiting an induced vulnerabilities, such as induction of senescence and cell death, for effective interrogation of HCC at early stage^6^.

Increasing evidence indicates that there are several forms of cell death including apoptosis, necrosis and oncosis etc^6^. Oncosis was identified by Friedrich von Recklinghausen more than a century ago, which exhibited almost opposite morphological phenotypes of apoptosis^7^. Apoptosis is hallmarked by characteristics of cell shrinkage and nuclear fragmentation while oncosis displays cell swelling and coagulation of the cytoplasm. Oncotic cell death involves progressive plasma membrane injury involving 3 distinct stages^8,9^, which was initiated by plasma membrane permeability alteration due to injury in stage 1. This results in uncontrolled exchange of intracellular and extracellular ions and water which lead to cell swelling without gross alteration of permeability. In stage 2, the cell membrane becoming leaky to propidium iodide and trypan blue, which results in a non-selective increase in membrane permeability. In stage 3, the cells eventually lost physical barrier of the plasma membrane and viability. Since early necrotic cells lose plasma membrane integrity judged by the trypan blue uptake^10^, the exclusion of trypan blue and propidium iodide by oncotic cells indicates that oncosis is not representative of early necrosis. Thus, oncosis was a form of accidental cell death hallmarked by cellular swelling, blebbing and increased membrane permeability caused by an activation of calpain I and related cytoskeletal rearrangement^7,11,12^, while necrosis is initiated by inflammation signaling and RIPK activation. Our early studies show that *Helicobacter pylori* induced atrophic gastritis via oncotic process which involves in VacA-elicited plasma membrane permeability lost and an activation of calpain I due to the influx of extracellular calcium^13^. However, the precise mechanism of action underlying oncosis remains elusive. In addition, it was unclear whether oncosis could be used as an inducible vulnerability for interrogating tumor progression.

Recent studies show that limited proteolysis of cell matrix protein profibrillin generates a circulating peptide Asprosin which circulates at nanomolar levels and exerts its physiological function in liver via a G protein-elicited cAMP-PKA pathway^14^. Umbilical cord serum had been demonstrated to harbor a high concentration of biologically active components and growth factors for cell proliferation, migration and differentiation^15^. Recent study revealed that umbilical cord tissue-derived MSCs could suppress colitis-associated colorectal cancer by inhibiting chronic inflammation^16^. Hepatoma cells cultured in 2% human serum sustained growth arrest and presented a hepatocyte-like morphology^17^, suggesting that circulating peptides in human serum may rewire cancer cell plasticity.

In the current study, we tested the hypothesis that umbilical cord serum peptide can alter HCC cell plasticity and used peptidome analyses to identify an active peptide derived from glutamyltransferase(GGT). We demonstrate that this peptide acts directly on HCC cell plasma membrane and induces an oncosis-like process. The elevation of intracellular calcium and sodium induces microtubule depolymerization. These findings provide a novel niche to interrogate HCC progression using umbilical cord serum peptide-induced oncosis.

## Results

### Identification of a biologically active peptide from human umbilical cord serum

**Figure 1:**
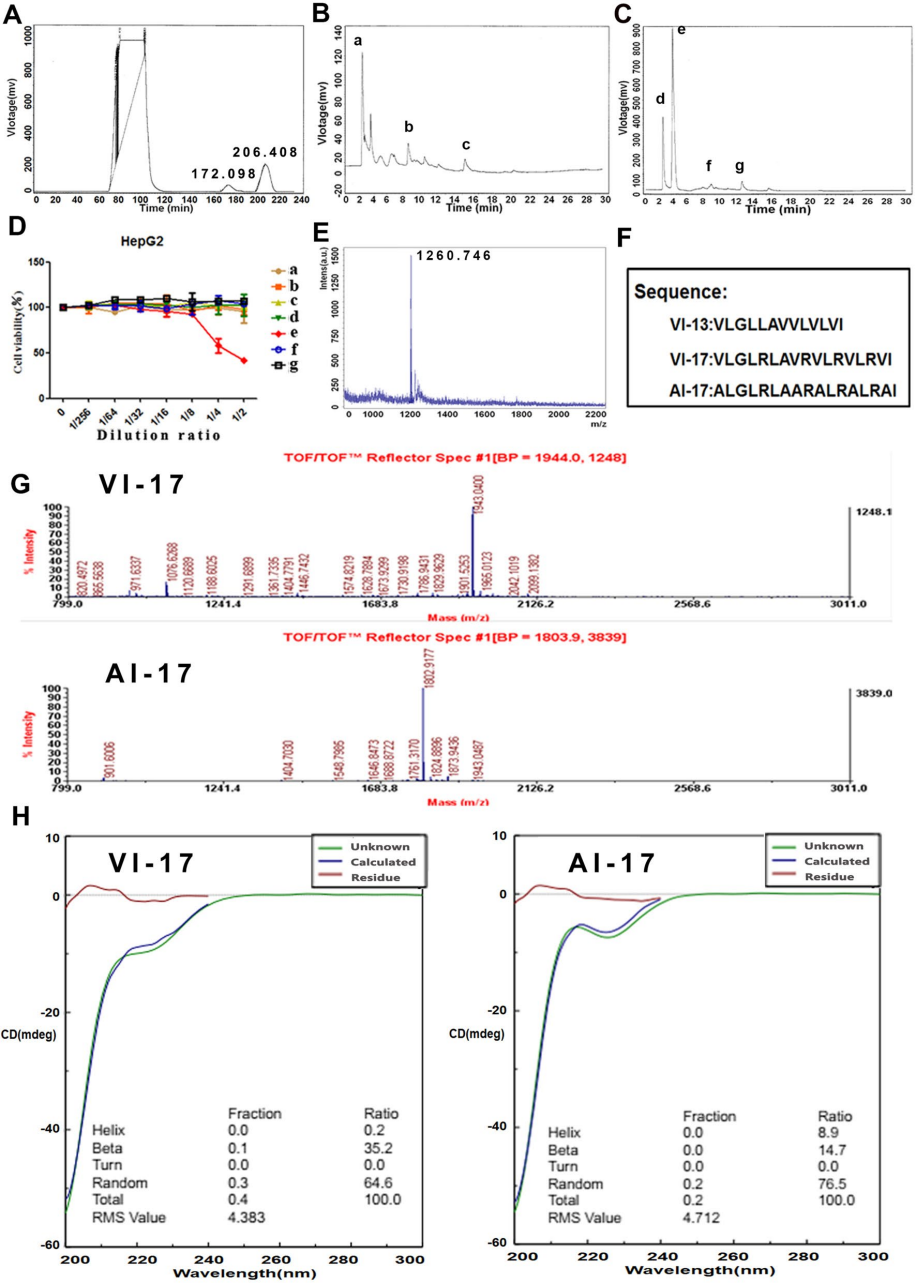
Identification of highly abundant peptide in human umbilical cord serum and the modification. **(A)** Size Exclusion Chromatograph of human umbilical cord serum. **(B,C)** Reversed phase liquid chromatography for the samples from A. **(D)** Bioactivity analysis for samples isolated from B–C. **(E)** Mass spectrometry analysis for sample e from B–C. **(F)** The amino acid sequences of peptides in this study. **(G)** TOF/TOF Reflect spectrum of VI-13, VI-17 and the control peptide AI-17. **(H)** CD spectra for VI-17 and the control peptide AI-17.

Human umbilical cord serum was isolated by SEC, and the chromatogram was shown in Fig. 1A. Two peaks were obtained at 172.098 min and 206.406 min, and were collected and offline separated again with reverse phase high pressure liquid chromatography in second dimension separation according to the time point, respectively (samples a–c in Fig. 1B were isolated from peak of 172.098 min and samples d-g in Fig. 1C were isolated from peak of 206.406 min). We then detected the activity of each sample on hepatoma cells and Sample e was proved as an antitumor component (Fig. 1D). Sample e was then detected by MALDI-TOF MS and one peak was significantly distinct with respect to m/z (1260.746) and the peptide was then sequenced, named as VI-13 (Fig. 1E). As the charge and amphiphilicity are crucial for the bioactivity of peptide, we inserted four arginine into peptide VI-13 to construct an analog peptide VI-17 with modified solubility and charge based on the linguistic model^18^. Previous study reported that peptides containing only arginine and valine as prototype residues were capable of forming antimicrobial activity^19^. Thus, the analog peptide VI-17 may possesses higher bioactivity. The peptide with replacement of Val to Ala in VI-17 was constructed as the control and named as AI-17 (Fig. 1F). As detected using TOF/TOF™ Reflect spectrum, the peptides were synthesized correctly with molecular weight of 1943.04 Da and 1802.91 Da, respectively (Fig. 1G). CD spectra for two peptides showed higher proportion of β-sheet in VI-17 than AI-17 (Fig. 1H).

### VI-17 peptide induced cell death in variety of cancer cell lines

**Figure 2:**
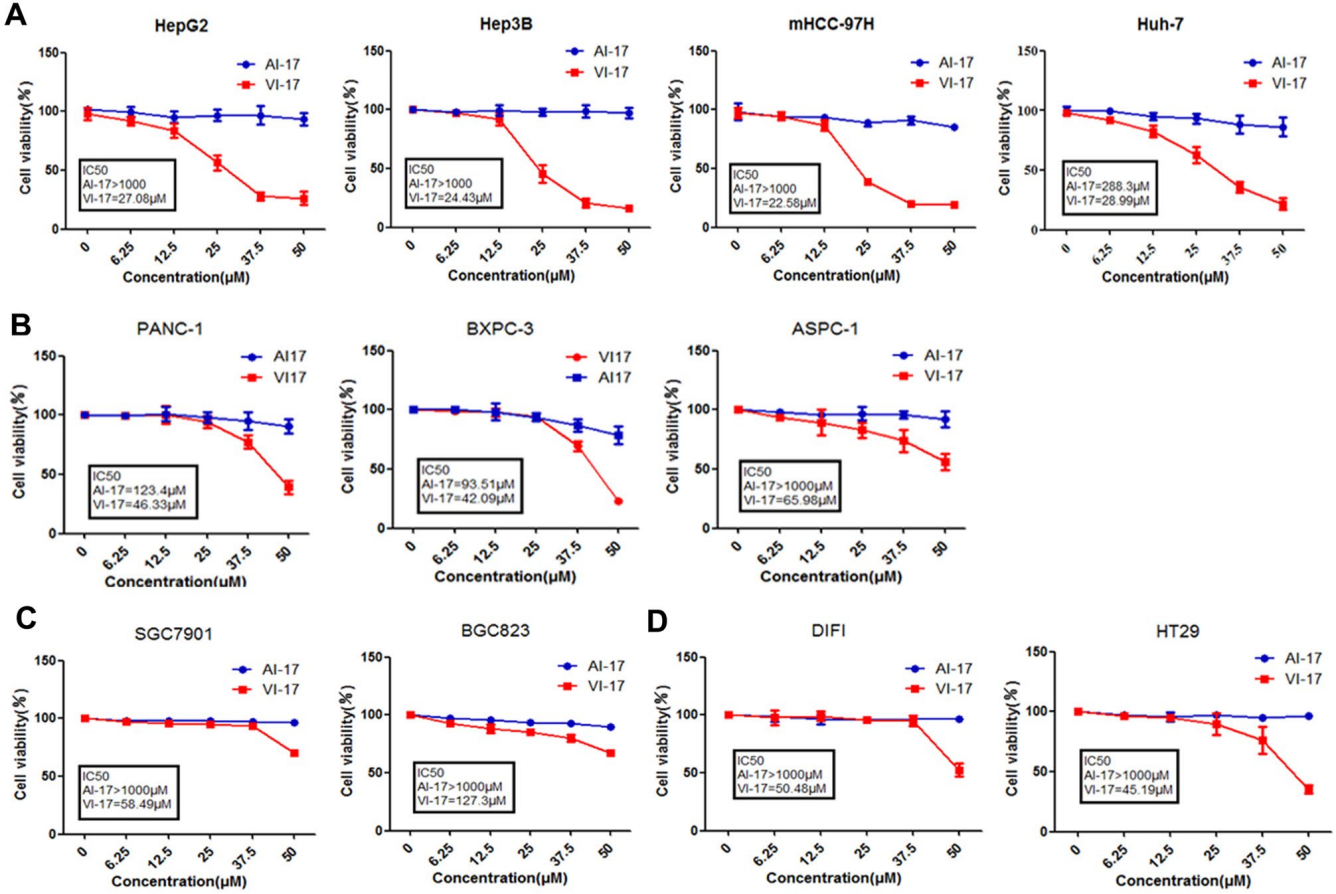
VI-17 was effective against multiple cancer cell lines in vitro. **(A)** MTT assays of the bioactivity of VI-17 against hepatoma cells. **(B)** MTT assays of the bioactivity of VI-17 against pancreatic cancer cells. **(C)** MTT assays of the bioactivity of VI-17 against gastric cancer cells. **(D)** MTT assays of the bioactivity of VI-17 against colonic cancer cells. Data are expressed as Mean ± SD of three independent experiments in triplicates.

Multiple cell lines, including hepatic cancer, pancreatic cancer, gastric cancer and colorectal cancer, were used to confirm the killing activity of VI-17 judged by MTT assay as reporter of cell viability. We found that VI-17 induced cell death process in several cancer cell lines in a dose-dependent manner. Cell lines derived from HCC exhibited a greater sensitive to VI-17 peptide (IC_50_ value: HepG2, 27.08 μM; Hep3B, 24.43 μM; mHCC9H, 22.58 μM; Hu-7, 28.99 μM) (Fig. 2A). In the same testing condition, the IC_50_ values were relatively higher for cell lines derived from pancreatic cancer (IC_50_ value: PANC-1, 46.33 μM; BXPC-3, 42.09 μM; ASPC-1, 65.98 μM) (Fig. 2B). The IC_50_ values were even higher for the cell lines from gastric cancer (IC_50_ value: SGC7901, 58.49 μM; BGC823, 127.3 μM) (Fig. 2C) and colon cancer (IC_50_ value: DIFI, 50.48 μM; HT-29, 45.19 μM) (Fig. 2D). However, the control peptide AI-17 exhibited less membrane permeabilizing activity to the aforementioned cell lines. Thus, we conclude that VI-17 peptide is a potent permeabilizing agent for HCC cell lines.

### Microtubule stabilizer Taxol attenuated VI-17-elicited cytotoxicity on HCC cells

**Figure 3:**
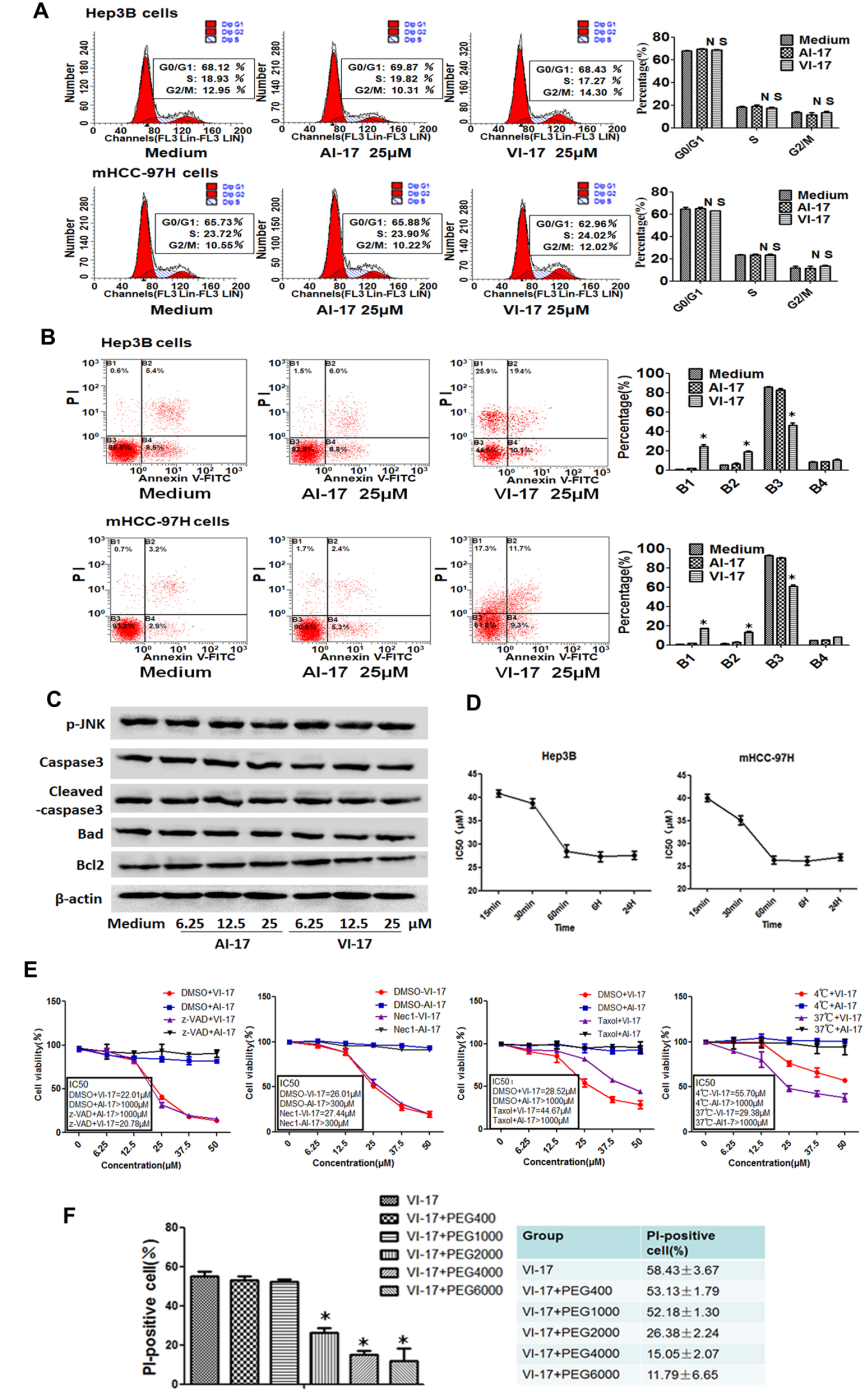
Taxol attenuated VI-17-elicited cytotoxicity on HCC cells. **(A)** Flow cytometric analysis for the effect of VI-17 on cell cycle. **(B)** Flow cytometric analysis for the effect of VI-17 on cell apoptosis. **(C)** Western blot analysis for expression of apoptotic molecules. **(D)** IC50 values of VI-17 on hepatoma cells at indicating time points according to MTT assays. Data are expressed as Mean ± SD of three independent experiments in triplicates. *p < 0.05. **(E)** MTT assays for the effects of z-VAD-fmk, necrostatin-1, taxol and low temperatures on VI-17 induced cell death of Hep3B cells. **(F)** Analysis for the ratio of PI-positive cell under PEGs pretreatment in VI-17-treated Hep3B cells. Data are expressed as Mean ± SD of three independent experiments in triplicates. *p < 0.05.

After characterization of VI-17 peptide in altering the membrane permeability in various cancer cell lines, we selected two representative cell lines, Hep3B and mHCC97H, for further investigation. To understand the mechanism of action underlying the VI-17-elicited cell fate change in HCC cells, we carried out flow cytometric analyses to determine the effect of VI-17 on the cell cycle and cell death. Surprisingly, VI-17 treatment resulted in no significant effect on cell cycle progression and apoptosis, compared to the control, but increased the proportion of necrotic cells (Fig. 3A,B). Western blot analyses also revealed that the protein levels of apoptotic proteins were not changed (Fig. 3C). The IC_50_ values judged by MTT assay at indicated time points showed that VI-17 induced cell membrane permeable in time-dependent manner. The IC_50_ value of VI-17 for Hep3B and mHCC-97H cells were 40.07 μM and 37.7 μM after 5 min of treatment, suggesting that VI-17 may induce rapid loss of cell membrane integrity (Fig. 3D).

To evaluate whether VI-17 induced cell injury and death via necrotic pathway, we pretreated cells with z-VAD-fmk, necrostatin-1 and taxol for 6 h before the addition of VI-17 peptide. MTT assays showed that the activity of VI-17 was not alleviated by neither z-VAD-fmk nor necrostatin-1 (Fig. 3E). Surprisingly, microtubule stabilizer taxol dramatically attenuated the effect of VI-17 on cell death (Fig. 3E). To test if VI-17 induced cell injury/death involves in membrane poration, we treated cells with peptides at 4 °C and 37 °C, respectively. After 5 min incubation, the treated cells were stained with MTT. As shown in Fig. 3E, VI-17 induced cell death occurred rapidly at 37 °C. The cell viability was not altered at 4 °C in the presence of VI-17 peptide.

Polyethylene glycol (PEG) has been widely used for grafting on the liposomes and offering a protective shell on the liposome surface. To test if PEG would protect cells from VI-17 peptide induced permeabilization effect, PEGs with diverse molecular weight were used to pretreat cells for 30 min followed by VI-17 treatment. As shown in Fig. 3F, the permeabilization effect of VI-17 was alleviated by PEGs with the molecular weight of 2 kDa or above. Thus, we conclude that VI-17 peptide induced membrane permeabilization and HCC cell injury independent of necrotic process.

### VI-17 peptide exhibited inhibition of HCC growth in vivo

**Figure 4:**
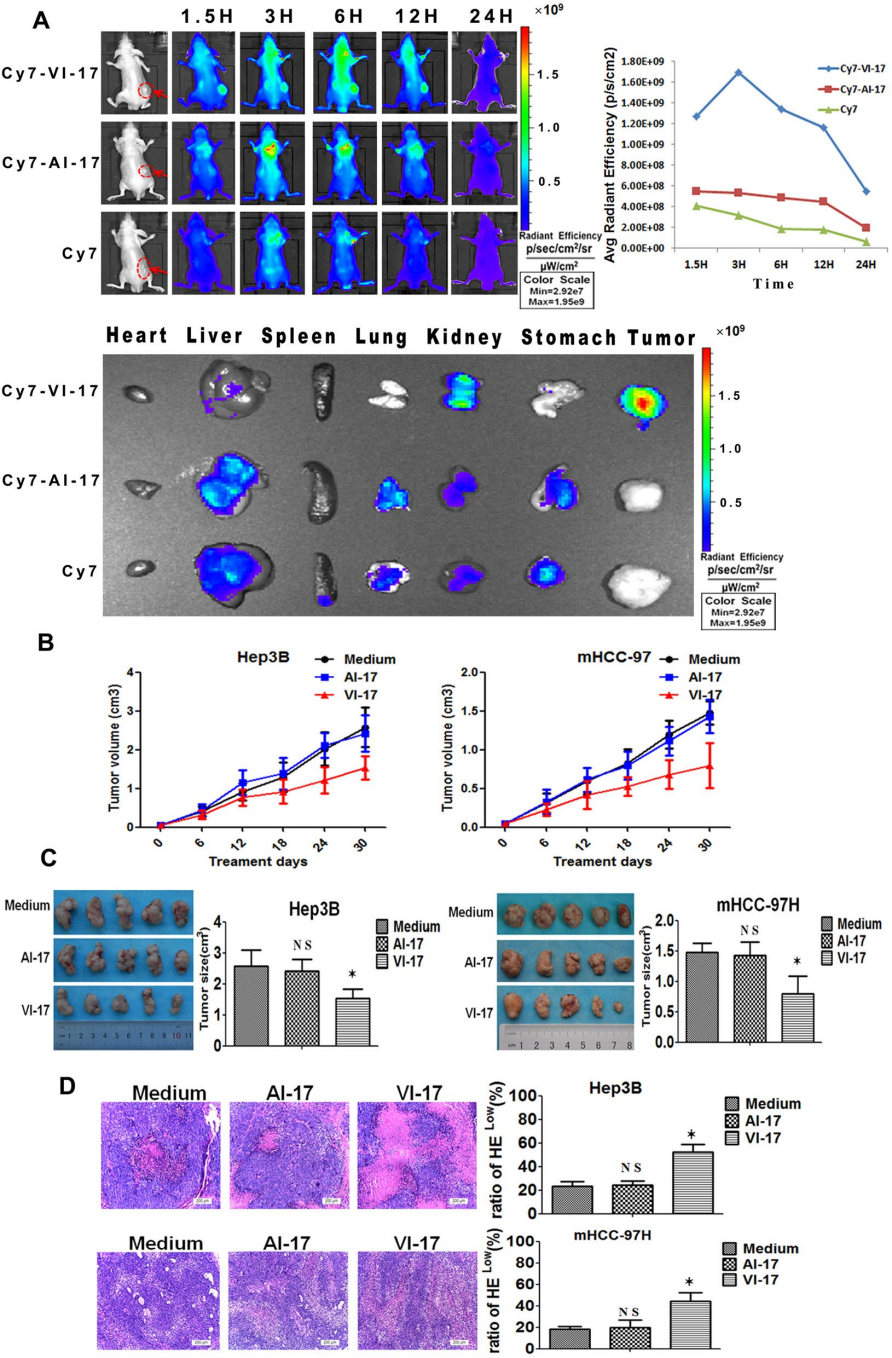
VI-17 was significantly accumulated in hepatoma tumor and exhibited anti-tumor activity in vivo and in vitro. **(A)** NIR-II fluorescence for tumor-bearing nude mice at indicating time points (left) and analysis of average radiant efficiency (right), NIR-II fluorescence for isolated tumor and organs (including heart, liver, spleen, lung, kidney and stomach). **(B)** Tumor volume evolution in tumor-bearing nude mice during the treatment period. **(C)** Analysis of tumor size in VI-17 treated tumor-bearing nude mice at 30 days. **(D)** HE staining for hemorrhagic necrosis area of *C*. Data are expressed as Mean ± SD from each group. *p < 0.05.

After evaluating the cellular effect of VI-17 peptide in cell culture, we sought to explore the peptide effect in animals. To this end, intravenous tail vein injection of VI-17 and control peptide (2 mg/kg) were performed on nude mice inoculated with Hep3B cells, and images were acquired at regular intervals over the course of 1.5 h, 3 h, 6 h, 12 h and 24 h, respectively. Tumor NIR fluorescence showed that the radiant efficiency of VI-17-Cy7 signal in the tumor increased and reached a maximum at 3 h, then reduced in the following period and eliminated at 24 h. However, the control peptide AI-17-Cy7 and medium-Cy7 group presented reduced signal patterns (Fig. 4A). The ex vivo fluorescence images of isolated tumor and organs at 12 h showed that the fluorescence signal was mostly concentrated in tumor, not in the normal organs (the lower panel).

To investigate the effects of VI-17 on HCC growth, tumor formation assays inoculated with Hep3B and mHCC-97H were performed in nude mice. Tumor growth curves based on volume showed that the treatment of VI-17 was effective to elicit tumor growth delay, compared with the control groups (Fig. 4B). Mice were then killed after treated with VI-17, AI-17 and medium for 30 days. As shown in **Fig. ****4****C**, the tumor volume in VI-17 treated mice were 1.52 ± 0.30 cm^3^ (Hep3B) and 0.80 ± 0.29 cm^3^ (mHCC-97H), which were significantly smaller than those of AI-17 treated animals (Hep3B: 2.42 ± 0.36 cm^3^, mHCC-97H: 1.43 ± 0.21 cm^3^). HE staining showed that the ratios of necrotic area in VI-17 treated mice were 52.5 ± 6.1% (Hep3B) and 44.1 ± 8.0% (mHCC-97H), the ratios of control groups were 24.1 ± 3.7% (Hep3B) and 20.0 ± 7.0%(mHCC-97H), indicating that VI-17 may induce persistent hemorrhagic necrosis (Fig. 4D).

### Ultra-structural characterization of VI-17 treated HCC cells

**Figure 5:**
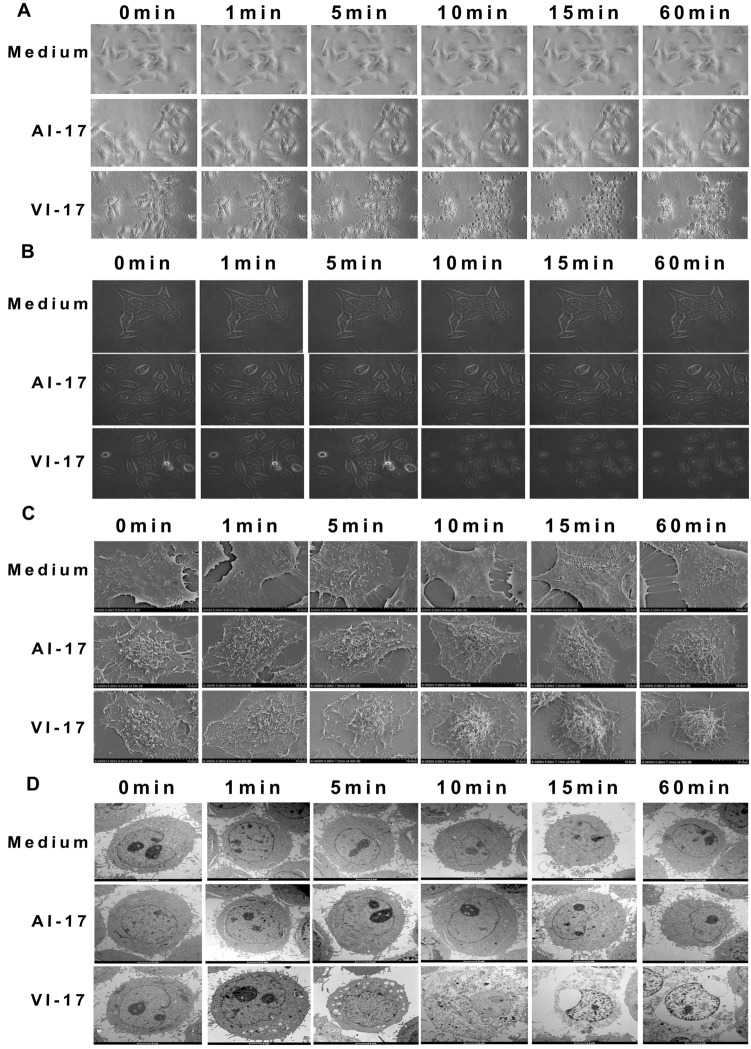
Morphological changes of VI-17 induced oncotic cell death. **(A)** Cell morphological changes observed by inverted microscopy. Original magnification: × 200. **(B)** Cell morphological changes observed by confocal microscopy at indicating time points. Original magnification: × 120. **(C)** Cell morphological changes observed by scanning electron microscopy at indicating time points. Original magnification: × 4000. **(D)** Cell morphological changes observed by transmission electron microscopy at indicating time points. Original magnification: × 6000.

HCC cells treated with VI-17 presented rapid cell morphological changes with a formation of blisters on cell membrane, which were devoid of major organelles and similar to that seen during oncotic cell death. These changes occurred in the majority of cells within 10 to 60 min of treatment with VI-17 (Fig. 5A,B).

To characterize the ultra-structural feature in VI-17-treated cells, scanning electron microscopic analyses were conducted to examine the structure changes of cell surfaces for peptide-treated cells. Specifically, pronounced clumping was readily apparent in VI-17-treated cells (Fig. 5C). Morphologically, formation of membrane blebs and surface wrinkles was observed with a loss of surface microvilli. Most importantly, heterogeneous pores were observed on the plasma membrane. However, cells treated with control peptide presented normal cell surface structures (Fig. 5C).

Transmission electron microscopy was also used to probe the ultrastructural changes of cells treated with VI-17. The mitochondria appeared to be less electron-dense as early as 1 min after treatment with VI-17, with the cristae lying further apart or not visible at 60 min. When cells were treated after 5 min, a number of peripherally placed vacuoles were observed within the cytoplasm, which was organelle-free. Nucleus remained integrated but dilated. All these morphological features including a translucent cytoplasm, disintegrated plasma membrane and swollen endoplasmic reticulum membranes, is a typical consequence of cell death caused by oncosis (Fig. 5D). Thus, we conclude that VI-17 peptide treatment induced HCC cell oncosis.

### VI-17 treatment led loss of intracellular ATP and lactate dehydrogenase leakage

**Figure 6:**
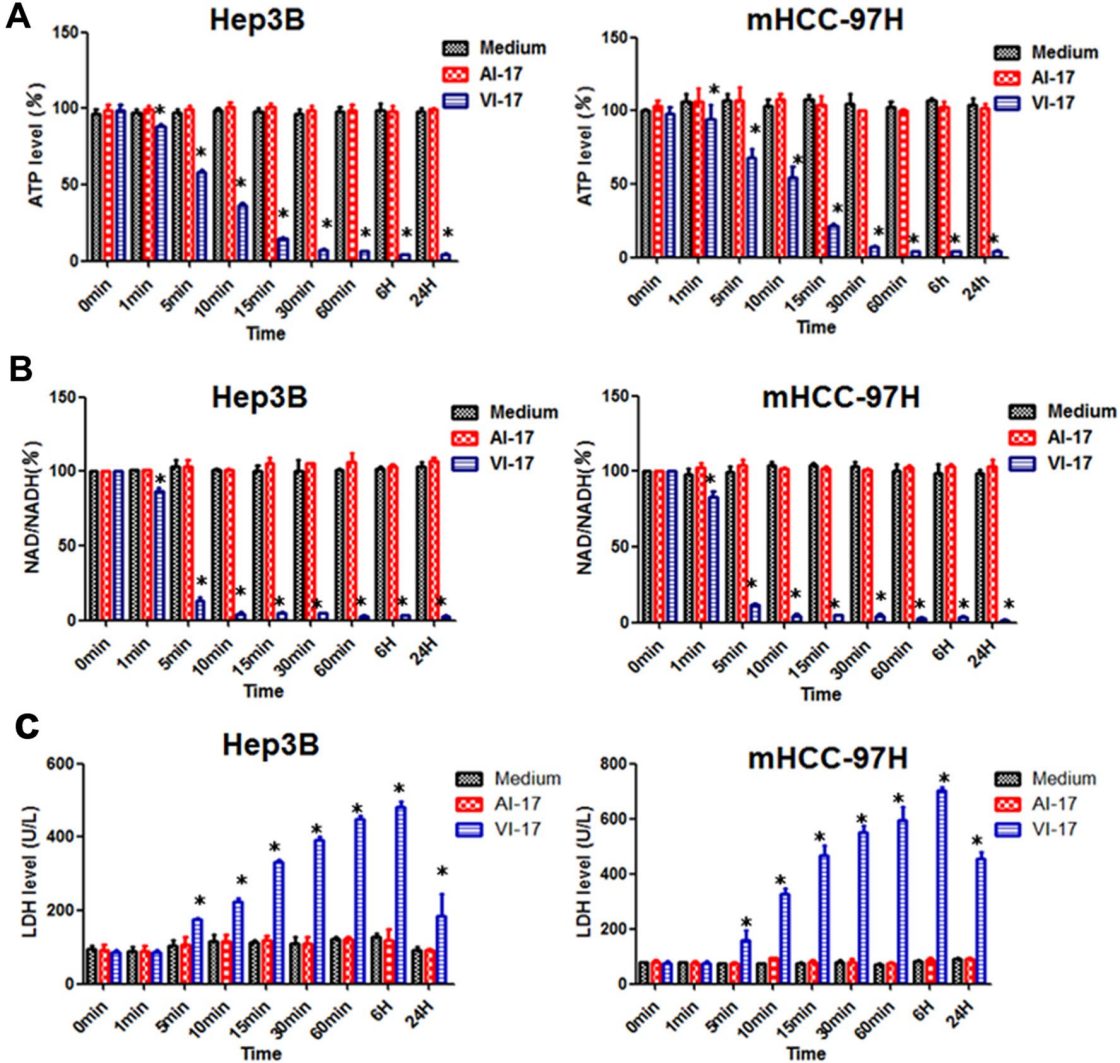
VI-17 treatment led loss of intracellular ATP and lactate dehydrogenase leakage. **(A)** Analysis of intracellular ATP level treated with VI-17 in Hep3B cells and mHCC-97H cells. **(B)** Analysis of the ratio of cellular NAD treated with VI-17 in Hep3B cells and mHCC-97H cells. **(C)** Analysis of the level of LDH in medium treated with VI-17 in Hep3B cells and mHCC-97H cells. Data are expressed as Mean ± SD of three independent experiments in triplicates. *p < 0.05.

As it was reported, the most crucial and early event of oncosis was membrane permeabilization and rapid loss of cellular ATP^20^. We therefore investigated whether VI-17 treatment could induce a prompt depletion of ATP. After treatment with VI-17 (25 μM), the intracellular ATP level decreased as early as 5 min and lowered to 10% of the control at 15 min, which is consistent with the time of cell blisters (Fig. 6A). As various metabolic perturbations contribute to loss of cellular ATP following NAD depletion, we detected whether the level of NAD was reduced. It was observed that the ratio of NAD/NADH sharply decreased as early as 5 min after the cells were exposed to VI-17 treatment, which was consistent with the temporal profile of ATP depletion in oncotic cells (Fig. 6B). We reason that the loss of intracellular ATP was not caused by consumption of NAD but release of ATP into extracellular medium due to an increased plasma membrane permeability elicited by VI-17 peptide. The release of LDH into extracellular medium would validate that VI-17-treated cells possessed ruptured plasma membrane. Thus, we evaluated the integrity of cell membrane by LDH release assay. As shown in Fig. 6C, the level of LDH in medium of VI-17-treated cells significantly higher than the control peptide treatment as early as 5 min and increased rapidly within 60 min.

### VI-17 treatment induced microtubule depolymerization

**Figure 7:**
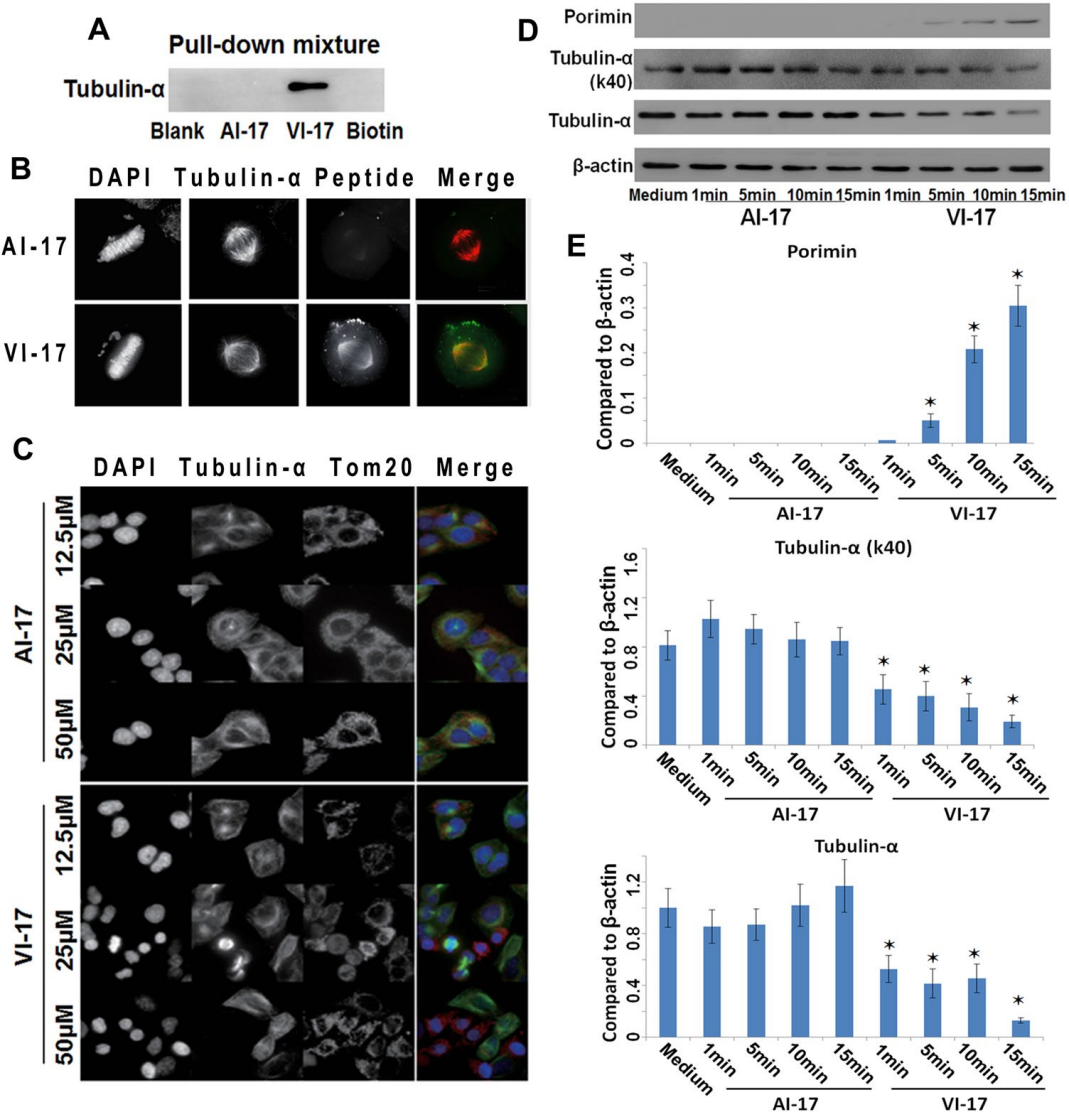
VI-17 interacted with tubulin-α, and induced tubulin-α degradation and porimin exposure. **(A)** Western blot analysis of candidate binding partners of VI-17 in pull-down mixture using biotin-VI-17 from Hep3B cells. **(B)** Immunofluorescence for cellular location of VI-17 and tubulin-α in VI-17 treated Hela cells. Original magnification: × 400. **(C)** Immunofluorescence for tubulin-α degradation and mitochondria swell in VI-17 treated Hep3B cells. Original magnification: × 200. **(D)** Western blot analysis of the expression of tubulin-α and porimin in VI-17 treated Hep3B cells at indicating time points. **(E)** Gray value analysis for the band in *D*. *p < 0.05.

To explore the underlying mechanism of VI-17 induced cell death, we performed pull-down assay to identify the binding targets of VI-17 in HCC cells. Although α-tubulin was recognized as a candidate binding partner of VI-17 (lane 3) in the initial immunoprecipitation experiment as shown in Fig. 7A, subsequent pull-down assay did not detect a direct interaction between VI-17 and α-tubulin. Previous studies have suggested that alterations of the cytoskeletal network are related to cell surface bleb formation and plasma membrane integrity. As cell blistering and nuclear morphology were observed in VI-17 treated cells, we therefore examined the subcellular distribution of VI-17. To visualize the distribution of VI-17 relative to α-tubulin, we performed cell immunofluorescence in HeLa cells (more proliferative and more infectious) and found that VI-17 (12.5 μM for 1 h) and α-tubulin were co-localized in the mitotic spindle (Fig. 7B), suggesting that VI-17 associates with microtubule cytoskeleton via some interacting proteins.

We next examined whether VI-17 peptide targets to other cellular structure such as mitochondria. Tom20 was characterized as the transit peptide receptor at the surface of the mitochondrion outer membrane, and enhanced staining of Tom20 was considered as the marker of mitochondria damage^21,22^. Treatment with higher concentrations of VI-17 at 25 μM and 50 μM induced tubulin depolymerization/degradation, accompanied with enhanced Tom20 exposure on mitochondria in Hep3B cells (Fig. 7C), indicating that mitochondria damage was involved in VI-17 induced oncosis. However, intact microtubules and normal mitochondria were observed in cells treated with control peptide. So we then examined the VI-17 induced reorganization of microtubule cytoskeleton by Western blotting analyses. The disruption of microtubule cytoskeleton integrity was evident by a dramatic decrease of α-tubulin protein in the membrane cytoskeleton fraction of VI-17-treated cells at the very early time of 5 min, which suggested that extracellular ion influx and dilution of intracellular ion concentration destabilized the microtubule cytoskeleton^13^. Consistent with this notion, the level of porimin, a membrane protein for sensing oncosis, was recruited to the plasma membrane fraction in a time-dependent manner (Fig. 7D). Our quantitative analyses from three independent experiments confirmed the significant correlation of increased level of porimin with decreased tubulin in membrane fraction of VI-17-treated cells (Fig. 7E). To visualize the potential internalization of porimin induced by VI-17, we carried out immunofluorescence microscopic analyses. Both VI-17 and control peptide treated cells were fixed after 15 min of treatment and stained for porimin. As shown in Supplementary Fig. 7F, the staining of porimin was much stronger in cytoplasm of VI-17 treated cells than that of control cells, suggesting that porimin has been internalized in response to VI-17 addition. Our real-time imaging of fluorescently labelled VI-17 confirmed its internalization (supplemental Fig. 7G). Thus, we conclude that VI-17 peptide elicited an oncotic process in HCC cells with hallmark of microtubule depolymerization and membrane protein internalization.

### VI-17 peptide induced an alteration of membrane permeability

**Figure 8:**
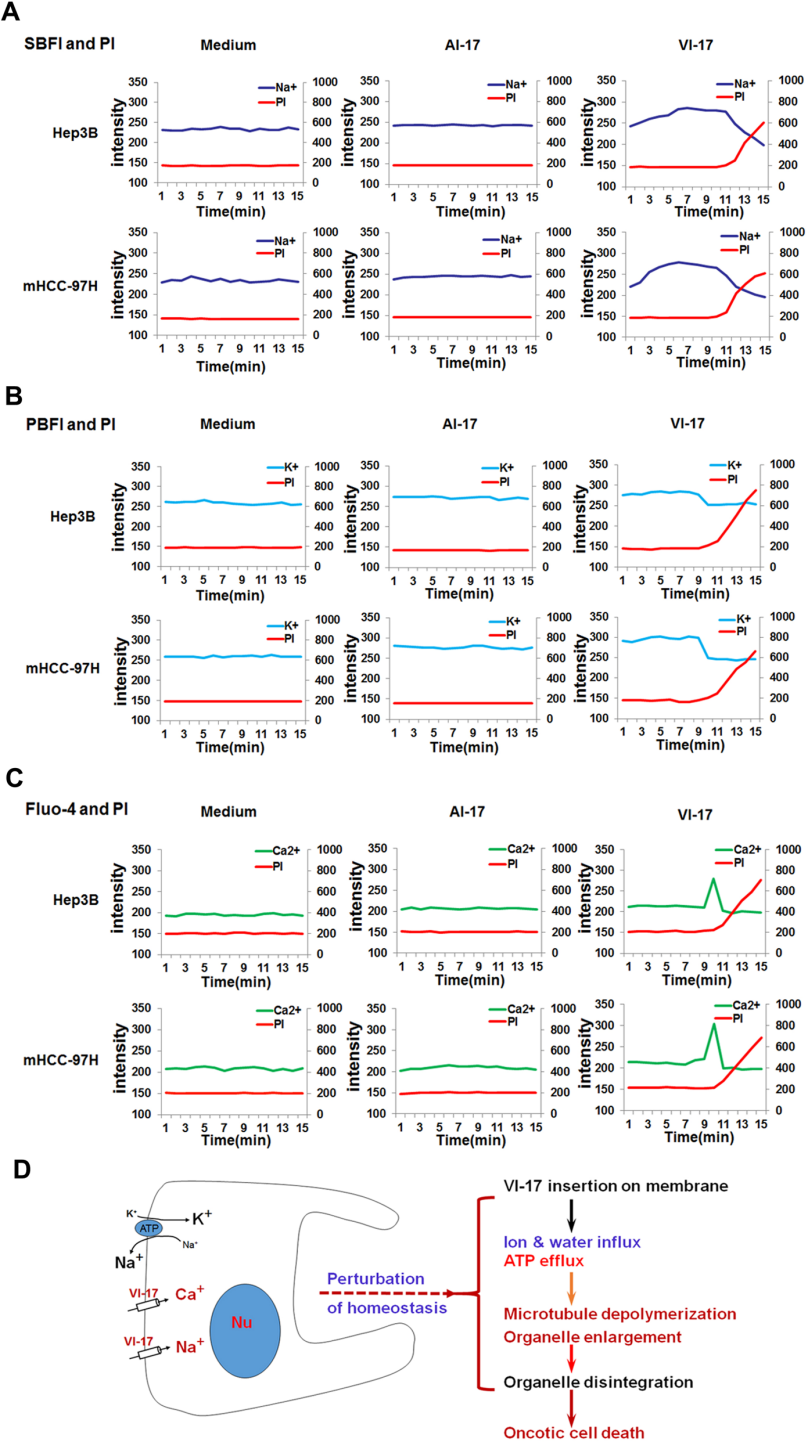
VI-17 rapidly induced selective leakage of sodium ion. **(A)** Analysis of the intensity of cellular Na^+^ signal using time phase microscopy. **(B)** Analysis of the intensity of cellular K^+^ signal using time phase microscopy. **(C)** Analysis of the intensity of cellular Ca^2+^ signal using time phase microscopy. **(D)** Cartoon for the dynamics of VI-17 elicited oncosis.

The ion influx/efflux was a driver for cell blisters and reorganization of cellular architect during oncosis. To further understand the mechanism of action underlying VI-17 peptide induced alteration of HCC cytoskeleton, we then examined whether the ion concentration was changed in cells in response to VI-17 treatment. Cells were labeled with the DNA binding fluorescent probe PI to distinguish necrotic cells from living cells as PI only entered cells when the plasma membrane is permeabilized. To this end, we measured intracellular sodium concentration in VI-17 peptide treated cells. As shown in Fig. 8A, there was a gradual rising intensity of intracellular sodium ion signal starting at 5 min after the peptide treatment of Hep3B cells. The similar rise of intracellular sodium was observed in mHCC-97H cells (Fig. 8A), indicating that the membrane permeability for sodium entry into HCC was increased by VI-17 peptide.

We next measured the intracellular potassium concentration. Consistent with the increased permeability in response to VI-17 treatment, the intracellular concentration of potassium decreased in both Hep3B and mHCC-97H cells at 9 min after the VI-17 peptide treatment (Fig. 8B). Consistent with our hypothesis of VI-17-induced permeability to ion exchanged across the plasma membrane, the intracellular calcium level was increased in both Hep3B and mHCC-97H cells at 9 min after the VI-17 peptide treatment (Fig. 8C). Thus, we conclude that the VI-17 peptide induced oncotic processes in HCC cancer cells via an increased membrane permeability to ion flux and macromolecules such as LDH (Fig. 8D).

## Discussion

Cell death was classified into three distinct cellular processes, apoptosis, ontosis and necrosis. Mounting evidence has delineated the molecular cascades underlying apoptosis and necrosis. Very little is known regarding the molecular mechanisms underlying oncosis despite the cytological characterization of oncotic hallmarks such as cellular swelling, blebbing and increased membrane permeability^7,23^. Recent studies show that tumor cell oncotic processes possess promising potential for an induced vulnerabilities. We have identified a biologically active peptide VI-17 from umbilical cord serum peptide which alters HCC cell plasticity via oncotic process. Mechanistically, the VI-17 peptide increased influx of extracellular calcium and sodium and efflux of potassium which elicited a membrane cytoskeletal reorganization (Fig. 8D). These findings provide a novel niche to interrogate HCC progression by eliciting oncotic processes.

Recent studies show that pharmacological activation of TRPV4 resulted in reduced tumor growth in breast cancer through oncosis and apoptosis^24^. Programed oncosis of ST2L-positive low-metastatic cells induced by interleukin-33 in tumor microenvironment of lung cancer led to a slower tumor growth^25^. Oncosis can be induced by severe cell injuries, such as complete ischemia, chemical toxins or the pro-oncosis receptor^26–28^. In the present study, the analog peptide VI-17, which was derived from the signal peptide of γ-GGT, presented cytotoxic effect on hepatic carcinoma via the oncotic pattern. Signal peptides were initially suggested as the endoplasmic reticulum targeting sequences of newly synthesized secretory or membrane proteins, which then were usually cleaved off by signal peptidase. However, recent studies have disclosed the post-targeting functions of signal peptides. These findings in this study may provide a new design strategy of signal peptides for antitumor drug development.

Formation of small pores in cell membrane was considered as the typical alternations of oncosis^29,30^. Osmoprotectants, such as PEG with a diameter larger than that of the membrane pores, could prevent cell swelling by blocking membrane pores^31,32^. As it was observed, PEGs with molecular weights ≥ 2000 can block cell membrane blebs formation in this study, suggesting that the size of the cell membrane pores induced by VI-17 was between that of PEG1000 and PEG2000. Besides, our result also revealed that the cytotoxic effect induced by VI-17 was weakened at low temperature as an ATP involved process. We thought that cell oncosis in this study was mediated by formation of small membrane pores, probably accompanied with the internalization of peptide VI-17.

Oncotic cell death was postulated to experience three stages during membrane injury, including ATP depletion with selective leakage of ions and water, non-selective increase in membrane permeability and eventual physical disruption of the cell membrane^8,33^. Previous studies have shown that rapid loss of cellular ATP may lead to deactivation of Na^+^-K^+^ ATPase at the cell membrane and result in excess Na^+^ influx, accompanied by water influx and a rapid increase in Ca^2+^^33,34^. Overload of intracellular calcium may cause hyperactivity of calpain-II, which may lead to irreversible injury of cell membrane and oncosis^35^. In this study, increased sodium influx was observed, with a sudden peak of cellular calcium when the membrane ruptured. Thus, we thought that sodium ion, accompanied with calcium, was involved in blebs formation induced by VI-17. Besides, sodium influx was occurred ahead of cellular ATP depletion in this study. We considered that this selectively acute imbalance of sodium ion here can be interpreted as the cause of loss of ATP induced by VI-17, which may lead to plasma membrane disruption.

Despite of the increasing evidences about the importance of oncosis on cell death and tumor development, the mechanisms underlying oncosis remain undetermined. Recent microarray analyses revealed that almost 2514 RNAs altered in oncosis, suggesting the involvement of multiple biochemical or signaling pathways in oncosis^26,33^. Previous study revealed that cytoskeleton was an important target for surface bleb formation^36,37^. Cytoskeleton proteins could interact with epithelial ion channels and regulate the channel activity^38–40^. Depletion of cytoskeleton-associated proteins can break the membrane-cytoskeleton system, leading to bleb formation and increased membrane permeability^41,42^. In this study, α-tubulin was suggested as a target of peptide VI-17 and α-tubulin depolyermization was observed at the beginning of oncotic processes, and the stabilization of microtubule by taxol pretreatment would weaken the depolymerization effect of VI-17 treatment. As α-tubulin was not detected in further pull-down assay (data not shown), the interaction of α-tubulin and VI-17 may be an outcome of microtubule depolymerization, but not the initiator for oncosis. We considered that VI-17 peptide permeabilized plasma membrane which resulted in influx of extracellular ions and depolymerization of microtubule network, and depolymerization of microtubule as the trigger of membrane fragmentation further accelerated the influx of sodium and calcium ions. Our study also revealed that VI-17 can induce a prompt recruitment of porimin to the plasma membrane. Porimin has been identified as a cell surface receptor involved in sensing oncosis^27,43^. However, our studies do not approve a direct interaction between porimin and VI-17 in this study. Thus, it would be of great interest in the future study to delineate how porimin coordinates with VI-17 peptide in oncotic process and whether their cross-talk can be used for an induced vulnerabilities in HCC interrogation using organoids model^52^.

In sum, we have identified a novel peptide VI-17 capable of inducing oncosis in HCC cells and uncovered the mechanism of action underlying VI-17-elicited oncosis. The peptide VI-17 would serve as a novel lead peptide for interrogation of HCC progress via eliciting oncosis. It will be worthwhile to investigate whether a combination of immunotherapy with pro-oncosis-based therapy can synergize the cytotoxic potential of recruited immune cells in HCC for better efficacy in clinical oncology.

## Materials and methods

### Collection of human umbilical cord serum

Umbilical cord serum was obtained immediately after fetus was born from five pregnant women, and was standing for 30 min at room temperature. The umbilical cord serum was centrifuged at 1200 rpm for 15 min and the supernatant was collected and stored at - 80 °C. The informed signed consents were obtained from patients. This project was approved by the institutional ethics committee of Xijing hospital and all procedures were carried according to the institutional guidelines of Xijing hospital.

### Size-exclusion chromatography

300 μL serum samples from five pregnant women were injected into the column packed with Superdex 75 gel equilibrated with mobile phase (0.020 mM KH_2_PO_4_ + 0.10 mM NaCl, pH 7.0), and the isocratic elution was operated. The flow rate was 0.5 mL/min and the detection wavelength was 280 nm. The fractions were collected and lyophilized.

### Reversed-phase liquid chromatography

The reversed-phase liquid chromatography was performed as previously described^44^. The mobile phase for reversed-phase liquid chromatography (RPLC) consisted of solution A (100% H_2_O, 0.1% Trifluoroacetic acid (TFA)) and solution B (100% CH_3_CN, 0.1% TFA). A 1.0 mL protein sample obtained from size-exclusion chromatography (SEC) was directly injected into an RPLC column equilibrated with mobile phase A by cumulative sampling, and then eluted with a linear gradient from 100% of solution A to 100% of solution B for 30 min with a delay for 10 min. The flow rate was 1.0 mL min^-1^ and the detection wavelength was 280 nm. The fractions were collected and lyophilized.

### Mass spectrometry analysis

MALDI-TOF mass spectrometry analysis of the fraction was performed previously^44^.

### Peptide modification

As it was predicted online (http://pepcalc.com/), the estimated molecular weight of VI-13 was 1320.75 Da, iso-electric point was pH 3.6 and the net charge at pH 7.0 was 0. As modification of known templates was a main approach for functional peptides design, we used peptide VI-13 as the template and modified the charge and amphiphilicity by inserting arginine into the peptide sequence and defined it VI-17. The estimated molecular weight of VI-17 was 1945.49 g/mol, iso-electric point was pH 12.58 and the net charge at pH 7 was 4.

### Circular dichroism spectra analyses

Circular dichroism (CD) spectra were recorded on a JASCO J-500A spectropolarimeter at room temperature (200–300 nm measure range; 0.5 nm data pitch; 1 mm cell length; 1 s D.I.T.; 1.0 nm band width; 50 nm/min scanning speed; and 2 accumulations). The peptides of high purity (> 98%) were purchased from Jier (Shanghai, China). The sample solutions were freshly prepared at a concentration of 1 mg/ml in Nacl, incubated at room temperature for 24 h and measured from 260 to 200 nm. All spectra were smoothed with the Spectra Manager Version2. The molar ellipticity [θ] was calculated using the molecular weight of the solute. The ellipticity was reproducible within an error of ± 5%.

### Cell lines

The cell lines (HepG2, Hep3B, mHCC9H, Huh-7, PANC-1, BXPC-3, ASPC-1, SGC7901, BGC823, DIFI, HT-29) used in this study were purchased from American Type Culture Collection (ATCC) and were cultured at 37 °C in 5% CO_2_ in DMEM/high glucose medium (Hyclone, Utah, USA) supplemented with 10% heat-inactivated fetal bovine serum (Biological Industries, Israel) as previously reported.

### Cell viability assays

Cells were plated in a 96-well plate (3000 cells/well) and were then treated with peptides, respectively. After incubation for 24 h, 20 mL MTT (5 mg/mL) was added to each well. Four hours later, 100 mL dimethyl sulfoxide was added to each well, and absorbance was measured at 570 nm using a Spectra Macplus microplate reader. Z-vad-fmk and Nec-1 were purchased from Abcam and were dissolved in DMSO. Cells were pretreated with Z-vad-fmk (30 μM), Nec-1 (10 μM) and taxol (10 nM) for 6 h, and then were incubated with the VI-17 peptides. For temperature test, cells were treated with peptides at 4 °C and 37 °C, respectively. For PEGs test, cells were pretreated with PEGs (30 mM) for 0.5 h, and then were incubated with peptides. Cell viability was set as the ratio of peptide-treated OD value divided by the medium group.

### Flow cytometry analysis for cell cycle and necrotic cell death

Cells were exposed to peptides (25 μM) for 24 h. For cell cycle analysis, cells were harvested and fixed in 70% cold ethanol overnight at 4 °C, and then incubated for 1 h in PBS containing 100 μg/ml RNase (Sigma) and 50 μg/ml PI. The DNA content was modeled according to the manufacturer's instructions. For necrotic cell death analysis, cells were harvested (including detached cells), and Annexin V-FITC staining and PI staining were performed according to the protocol. Cells with positive Annexin V and negative PI staining were classified as early apoptotic cells; cells with both positive PI and Annexin V staining were classified as late apoptotic cells; cells with both negative PI and Annexin V staining were classified as live cells; cells with positive PI and negative Annexin V staining were classified as necrotic cell.

### Fluorescence imaging in xenograft tumor models

Hep3B cells (1 × 10^7^ cells) were harvested and resuspended in 50 mL DMEM medium and injected subcutaneously into the right flank of 6-week-old female BALB/c nude mice. When tumor volumes reached approximately 50 mm^3^, the mice were randomly divided into three groups (n = 7 mice /group): VI-17-Cy7, AI-17-Cy7 and medium-Cy7. Mice were intravenously injected with VI-17-Cy7 and AI-17-Cy7 (8 mg/kg) or medium-Cy7 and were imaged in the NIR-II window at 1.5 h, 3 h, 6 h, 12 h, and 24 h, respectively. Tumor NIR fluorescence was performed in vivo and the radiant efficiency was calculated. All mice were maintained at an animal facility under pathogen-free conditions. Experimental procedures and handling of mice were carried out in accordance with experimental animal guidelines approved by our Institutional Animal Care and Use Committee. All experimental protocols were approved by the institutional ethics committee of Xijing hospital, including any details. The study was carried out in compliance with the ARRIVE guidelines.

### Antitumor efficacy in xenograft tumor models

A xenograft tumor model was established as reported previously^45^. Mice were intravenously injected with VI-17 and AI-17 (8 mg/kg) or medium once every 72 h. After 30 days, mice were sacrificed and tumors were excised for use in other assays. Experimental procedures and handling of mice were carried out in accordance with experimental animal guidelines approved by the institutional ethics committee of Xijing hospital. All experimental protocols were approved by the institutional ethics committee of Xijing hospital, including any details. The study was carried out in compliance with the ARRIVE guidelines.

### Inverted microscopy

Hep3B and mHCC-97H cells were seeded at 3000 cells/well in 6-well plates and allowed to grow for three days to optimize membrane structures in the culture. Cells were treated with VI-17 (25 μM) and cell morphological changes of cells were assessed by bright field of inverted microscopy at 100 × magnification at the indicating time points.

### Confocal microscopy

#### Live cell imaging of unlabeled cells.

Live cell imaging was performed as previous^46^. Briefly, hep3B and mHCC-97H cells were seeded at 5000 cells/well in complete medium in an 8-well chamber slide (Sigma) and were cultured overnight. Cells were washed twice with a serum-free RPMI, treated with 25 μM of peptide dissolved in RPMI and investigated using Bright Field on a Leica TCS SP5 confocal microscope. The microscope was equipped with an incubation chamber with CO_2_ and temperature control. All confocal imaging experiments were conducted at the indicating time, respectively.

#### Live cell imaging-propidium iodide and ion indicators.

Cells were plated onto a 35-mm glass bottom dish (Nest). Cells were treated with peptides (25 μM).Time-lapse microscopy for the intracellular SBFI, PBFI and Fluo-4 concentration were described previously^47^. Unprocessed images were analyzed by ImageJ software.

#### Fixed cell imaging

Cells were seeded into 4-well chamber slides (ThermoFisher Scientific) and incubated with peptides (25 μM) for 1 h, and then were fixed in 4% paraformaldehyde and blocked with 10% goat serum for 30 min. Cells were stained with primary and secondary antibodies as indicated. Cell nuclei were counterstained with DAPI. Laser confocal microscopy was performed, using an Olympus Fluoview FV 1000 confocal laser microscope (Olympus). Images were captured at 120 × magnification.

### Transmission electron microscopy

Cells were seeded at 1 × 10^5^ cells per well in 6-well plates and allowed to grow for three days to optimize membrane structures in the culture. Cells were then treated with peptides dissolved in serum-free RPMI at 25 μM. Cells were then washed with PBS before fixation for 24 h at 4 °C with 4% formal aldehyde and 1% glutaraldehyde in a HEPES buffer at pH 7.8. Dehydration and post-fixation protocols included incubation in a 5% buffered tannic acid and incubation in 1% osmium-reduced ferrocyanide. Ultrathin sections were prepared, and uranyl acetate (5%) and Reynolds’s lead citrate were used for staining and contrasting^13^. Samples were examined on a JEOL JEM-1010 transmission electron microscope, and images were taken with an Olympus Morada side-mounted TEM CCD camera (Olympus soft imaging solutions, GmbH, Germany).

### Assays for cellular ATP and NAD

We detected the intracellular ATP level using a commercial detection kit (Beyotime, Nantong, China) according to the manufacturer’s instructions. Luciferase activity was measured as the instruction of ATP level by Dual-Luciferase Reporter Assay System. Briefly, cells were plated in 24-well plates and treated with peptides at indicated time. The cells were washed with precooling PBS and lysed on the ice, centrifugated and the supernatants were collected. The samples were mixed with ATP detection solution and the RLU values were detected. For total NADH determination, cells were plated in 24-well plates and treated with peptides at indicated time. The cells were washed with precooling PBS and frozen-thawed twice. Cells were centrifugated and the supernatants were collected. For NADH detection, the supernatants were then bathed at 60 ℃ for 30 min to eliminate NAD. Every 50 μl samples were mixed with 98 μl NAD Cycling buffer + 2 μl NAD Cycling Enzyme Mix at room temperature for transition of NAD to NADH. The samples were then mixed with 100 μl NADH Developer at room temperature for 2 h, and were detected at 450 nm wavelength. The NAD ratio = (total NADH-NADH)/NADH.

### Assays for lactate dehydrogenase release

Cells were seeded in 24-well plate and were treated with peptides for indicated time. The medium was then centrifuged and collected. Extracellular level of lactate dehydrogenase was detected according to the protocol (Lactate dehydrogenase assay kit, Nanjing Jiancheng, China). Briefly, the medium was incubated with coenzyme I and 2.4-DNPH for 15 min at 37℃ respectively, then was incubated with 0.4 mol/L NaOH for 5 min at room temperature and was detected at 450 nm wavelength.

### Western blotting

Cell lysates were prepared in lysis buffer (150 mM Tris–HCl, 3% SDS, 0.15% bromophenol blue and 15% glycerol, pH 6.8). Samples were subjected to electrophoresis and transferred to polyvinylidene difluoride (PVDF) membranes by electroblotting. Immunoreactive proteins were visualized with ECL method (Pierce).

### Pull-down assays

The experiments were performed as previously described and summarized here^48–51^. Hep3B cells (1 × 10^6^) were lysed in 1 ml of lysis buffer (50 mM HEPES, pH 7.4, 150 mM NaCl, 0.1% Triton X-100, 1 mM EDTA, 10 μg/ml, aprotinin, 10 μg/ml leupeptin, 1 mM phenylmethylsulfonyl fluoride). Lysates were clarified by centrifugation at 16,000 × *g* for 10 min at 4 °C. Biotinylated peptides were binded to Streptavidin in spin columns. For immunoprecipitation, 0.4 ml lysate was incubated with 0.2 mg of biotin-VI-17 or biotin-AI-17 at 4 °C for 2 h. Spin columns were washed three times with 250 µl of lysis buffer containing 0.25 M NaCl. The precipitates were captured by 250 µl of Elution Buffer and were analyzed by standard immunoblot procedures. The immunoprecipitated proteins were subjected to SDS-PAGE and visualized by SimplyBlue SafeStain (Life technologies). Each band was trypsinized and subjected to MALDI-TOF MS analysis with MALDI-TOF/TOF5800 (AB SCIEX, Tokyo, Japan). These experiments were repeated thrice and identified proteins in at least two experiments were considered as candidates for peptide-binding partners. Precipitated proteins were analyzed by Western blotting.

### Statistical analysis

Statistical analysis was performed using the SPSS17.0 software and GraphPad Prism software Version 5. All experiments were performed three times. All results were displayed as a means ± SD and analyzed by Student’s t-test. p < 0.05 was considered statistically significant.

## Supplementary Information

Supplementary Information.
