# Supplementary material for: Potent antitumor activity of a glutamyltransferase-derived peptide via an activation of oncosis pathway
Source: Sci Rep. 2021 Aug 13;11:16507. doi: 10.1038/s41598-021-93055-5 (PMC8363616; doi:10.1038/s41598-021-93055-5)
Supplement: Supplementary file 1 — Supplementary Information. [file 41598_2021_93055_MOESM1_ESM.docx]

**Potent antitumor activity of a glutamyltraferase-derived peptide via an activation of oncosis pathway**

**Author list**: Cheng Fang^1,^*, Wenhui Li^2,^*, Ruozhe Yin^3,^*, Donglie Zhu^3^, Xing Liu^4^ , Huihui Wu^4^, Qingqiang Wang^3^, Wenwen Wang^4^, Quan Bai^5^, Biliang Chen^6^, Xuebiao Yao^4,ǂ^,Yong Chen^3,ǂ^

^1^Department of Hepatic Surgery IV, Eastern Hepatobiliary Surgery Hospital, Second Military Medical University, Shanghai 200438, China; ^2^Department of Gynecology and Obstetrics, Changhai Hospital, Second Military Medical University, Shanghai 200438, China;^3^Department of Hepatobiliary Surgery, Xijing Hospital, Fourth Military Medical University, Xi’an 710032*,* China; ^4^Anhui Key Laboratory for Cellular Dynamics and Chemical Biology and Hefei National Science Center, University of Science and Technology of China, Hefei, 230000,China; ^5^Key Lab of Modern Separation Science in Shaanxi Province, Northwest University, Xi’an 710069, China; ^6^Department of Gynecology and Obstetrics, Xijing Hospital, Fourth Military Medical University, Xi’an 710032, China;

* Cheng Fang, Wenhui Li, Ruozhe Yin and their institutions contributed equally to this work.

Correspondence:

Dr. Yong Chen; E-mail: [cheny@fmmu.edu.cn](mailto:yongchen62@yahoo.com)

Dr. Xuebiao Yao; Email: [yaoxb@ustc.edu.cn](mailto:yaoxb@ustc.edu.cn)

**Original blots of Figure 3C**

**
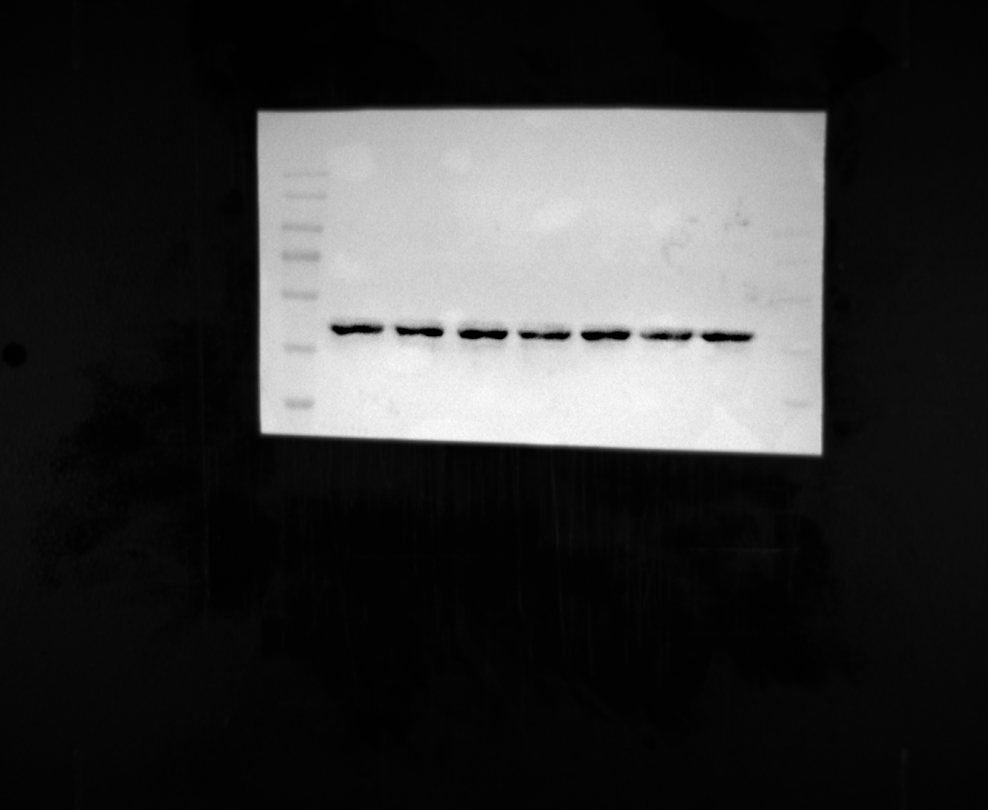
**

p-JNK

**
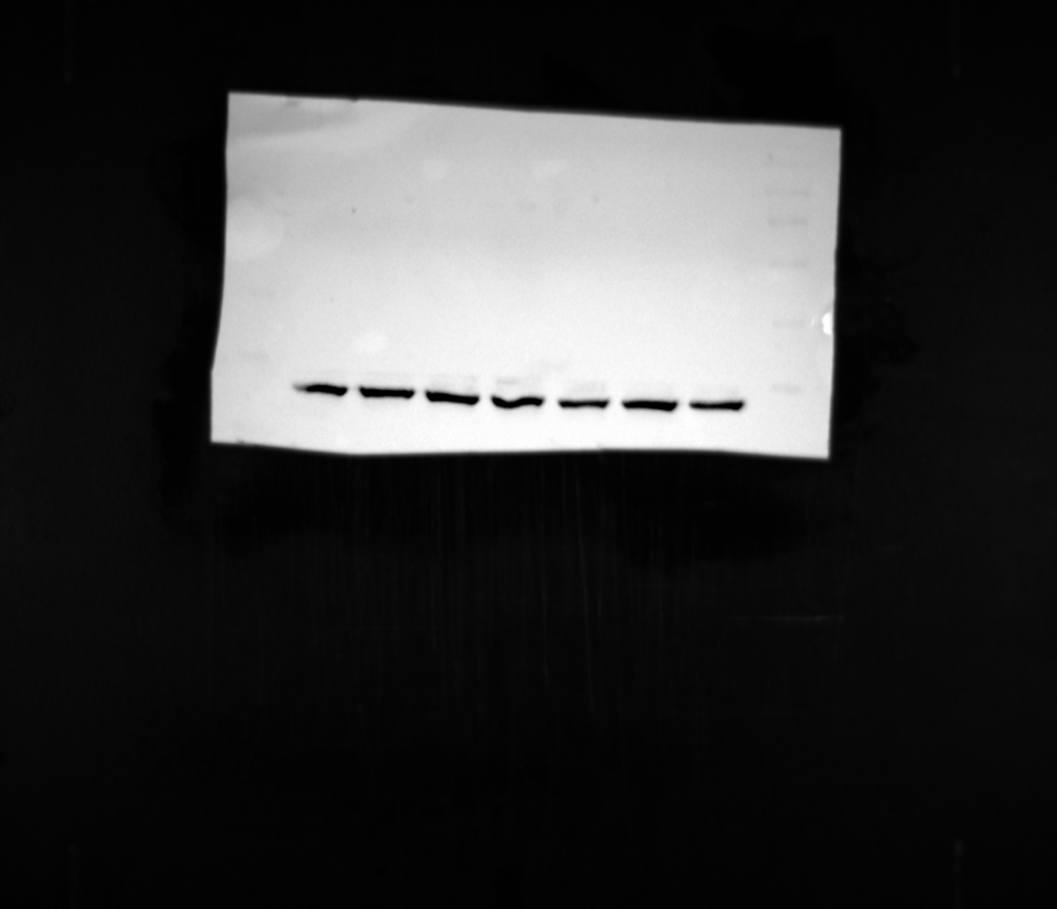
**

Caspase3

**
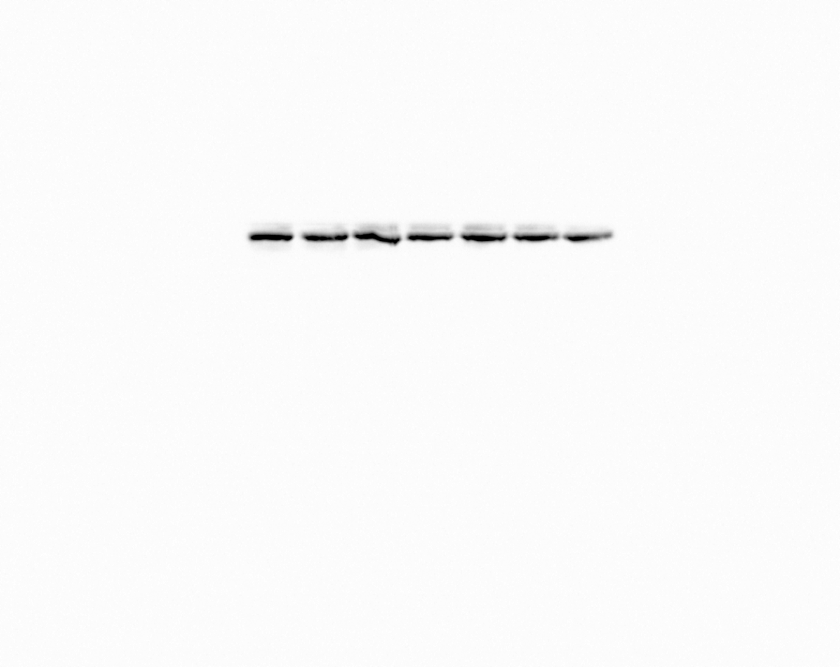
**

Cleaved-Caspase3

**
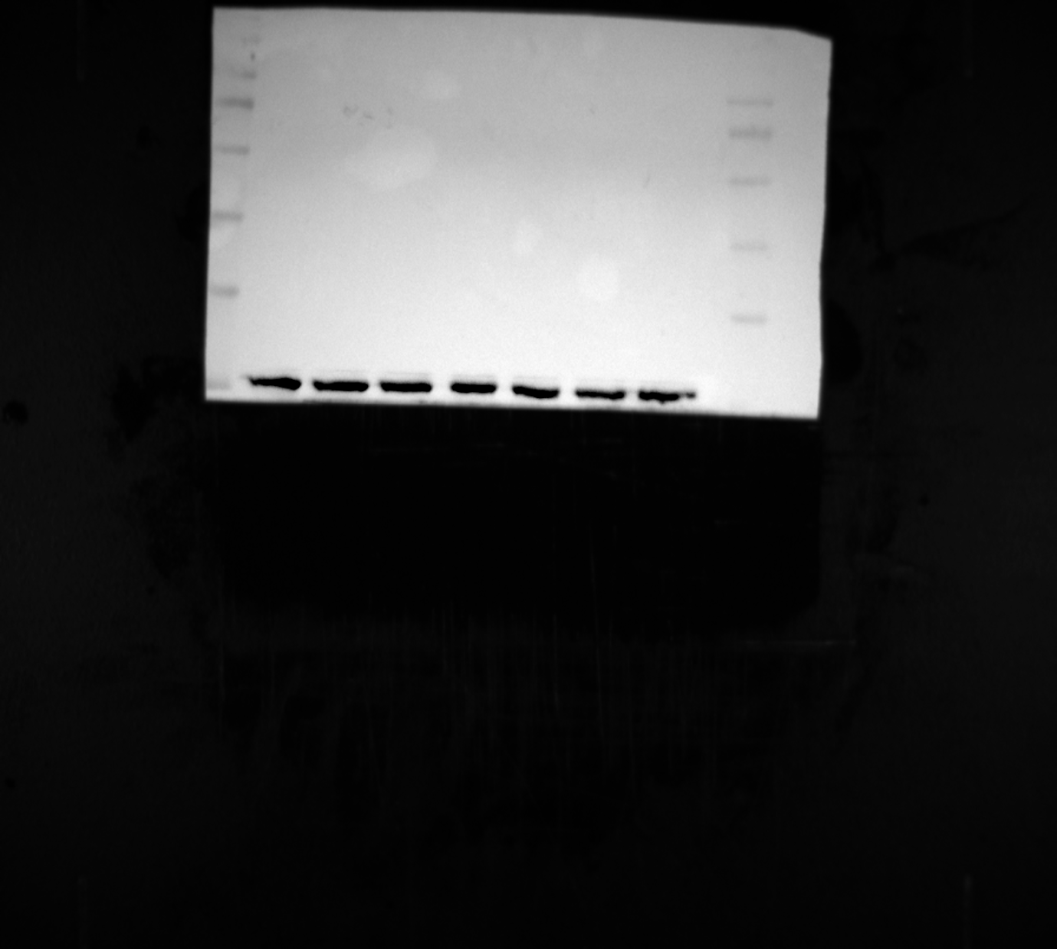
**

Bad

**
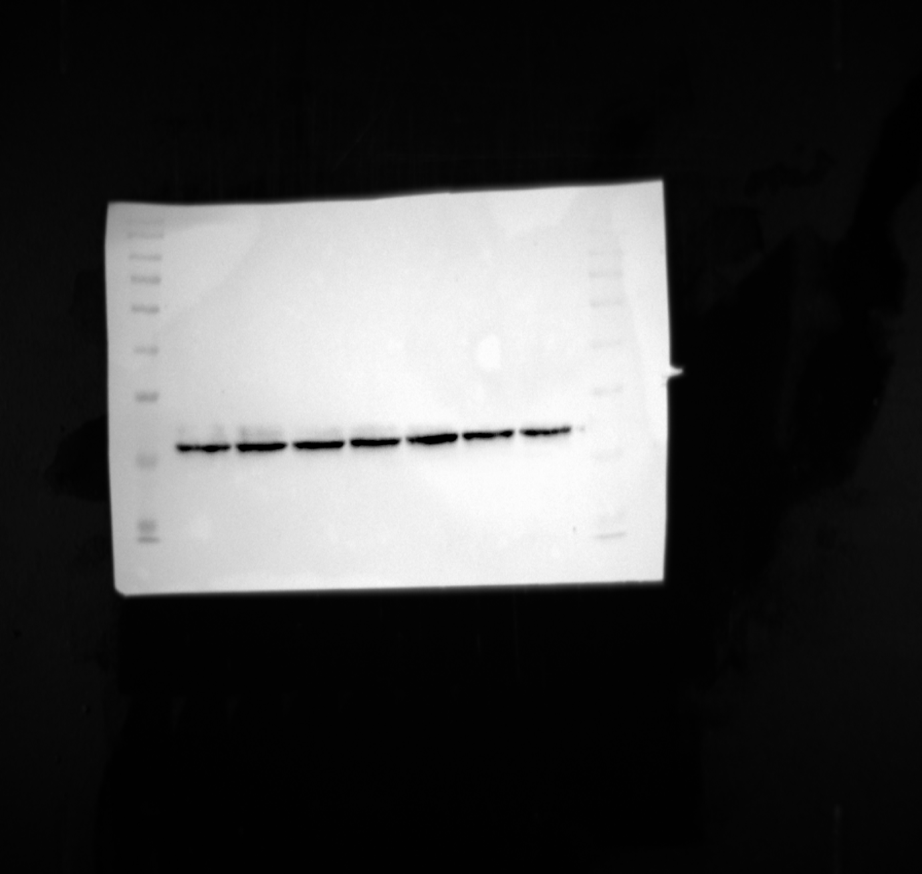
**

Bcl2

**
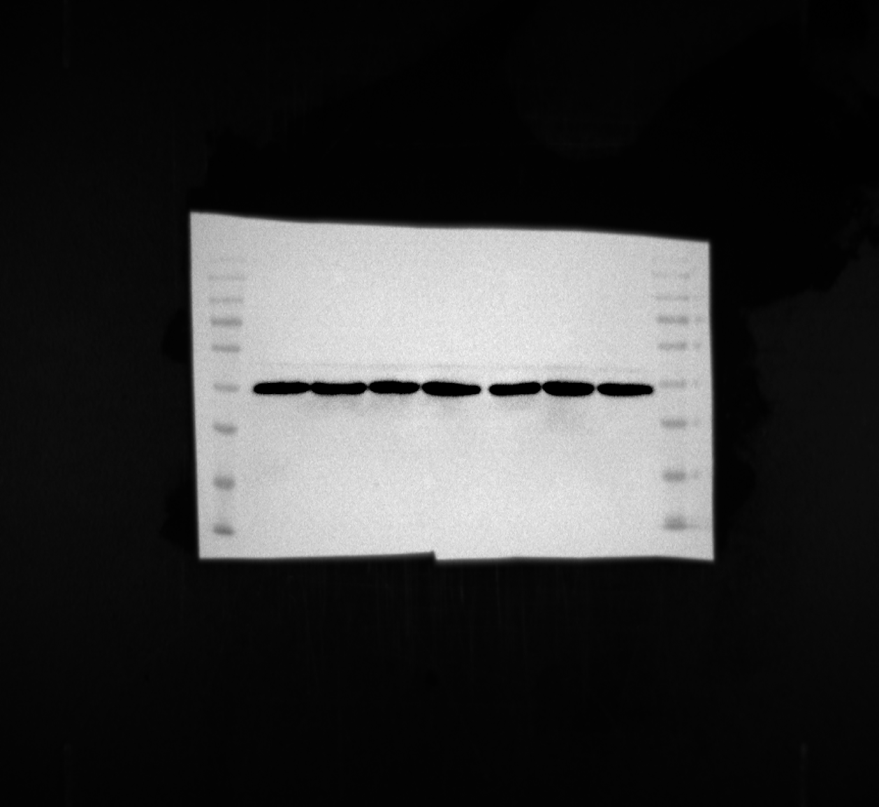
**

β-actin

**Original blots of Figure 7A**

**
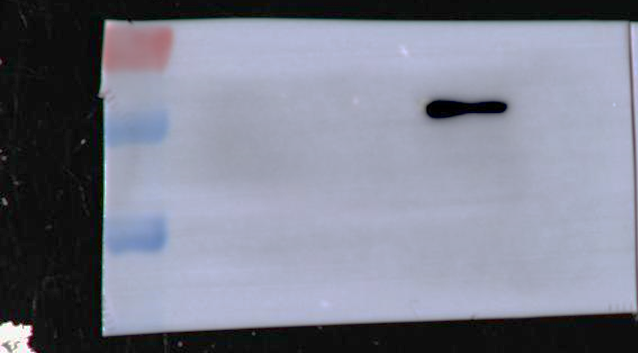
**

Tubulin-α

**Original blots of Figure 7D**

**
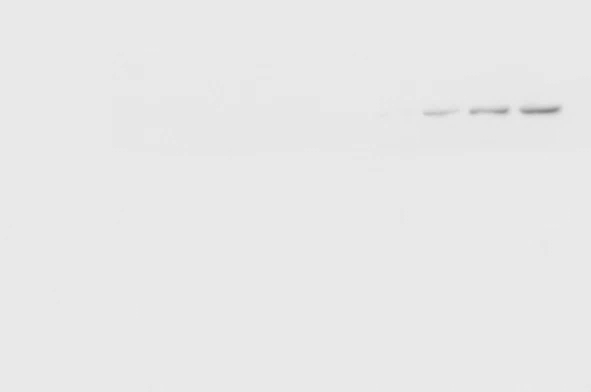
**

Porimin

Tubulin-α(k40)

**
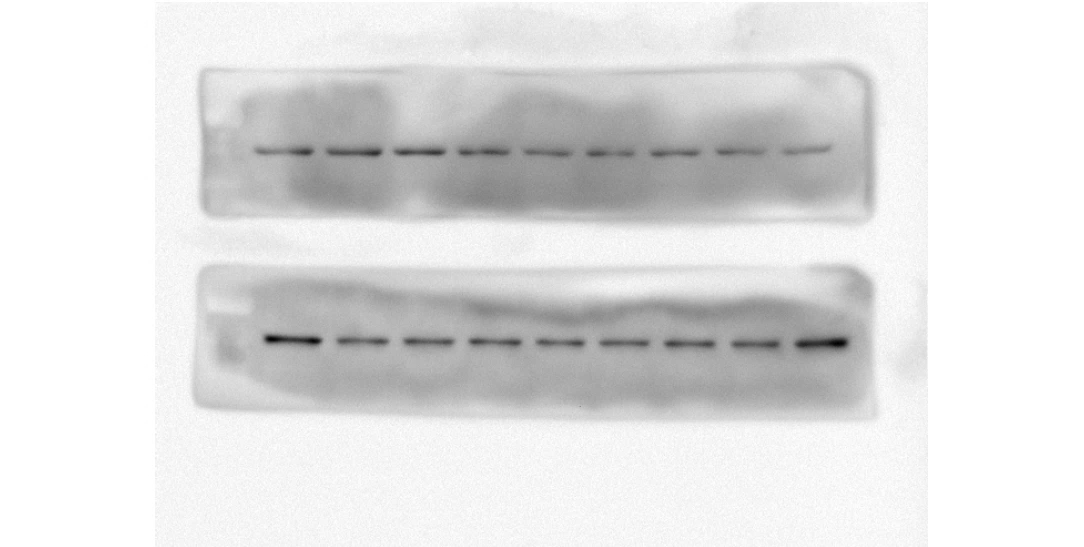
**

**
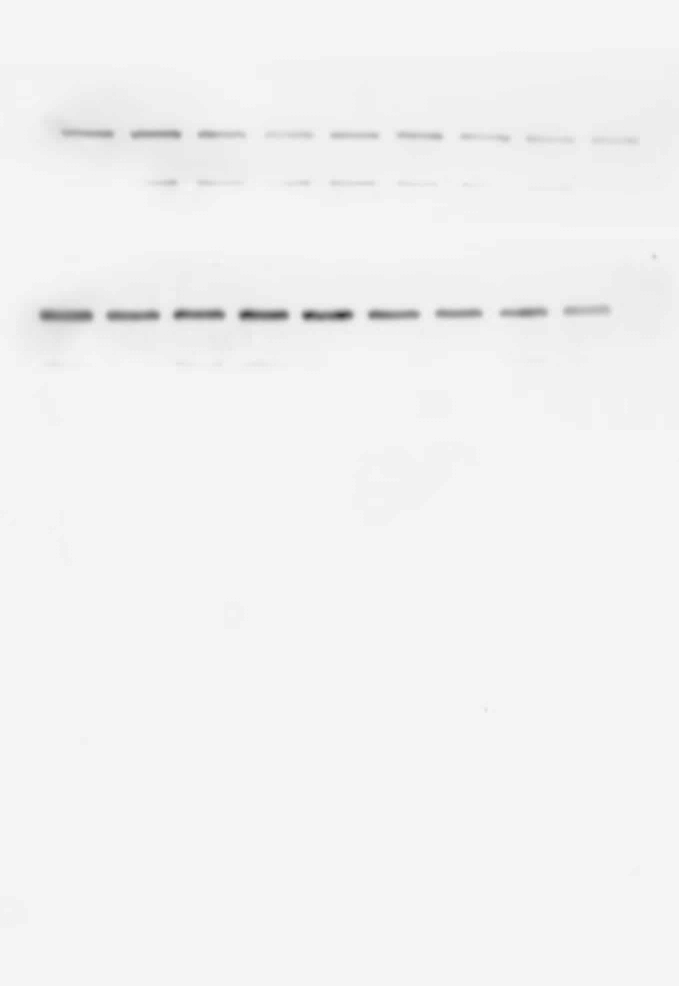
**

Tubulin-α

**
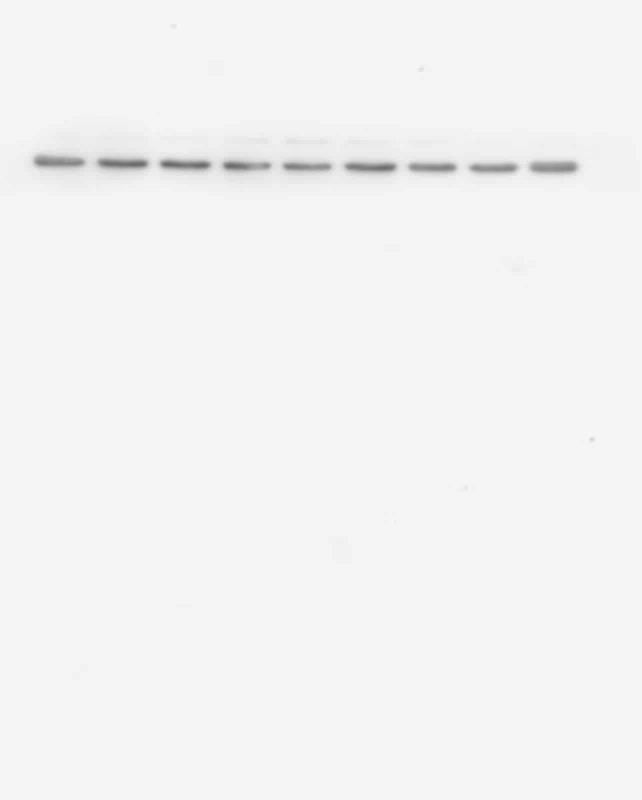
**

β-actin

**Supplementary Figure7 F** Immunofluorescence microscopic analysis of porimin in Hep3B cells induced by VI-17 for 15 min(anti-porimin dilution 1:200). Origin magnification: ×100.

**
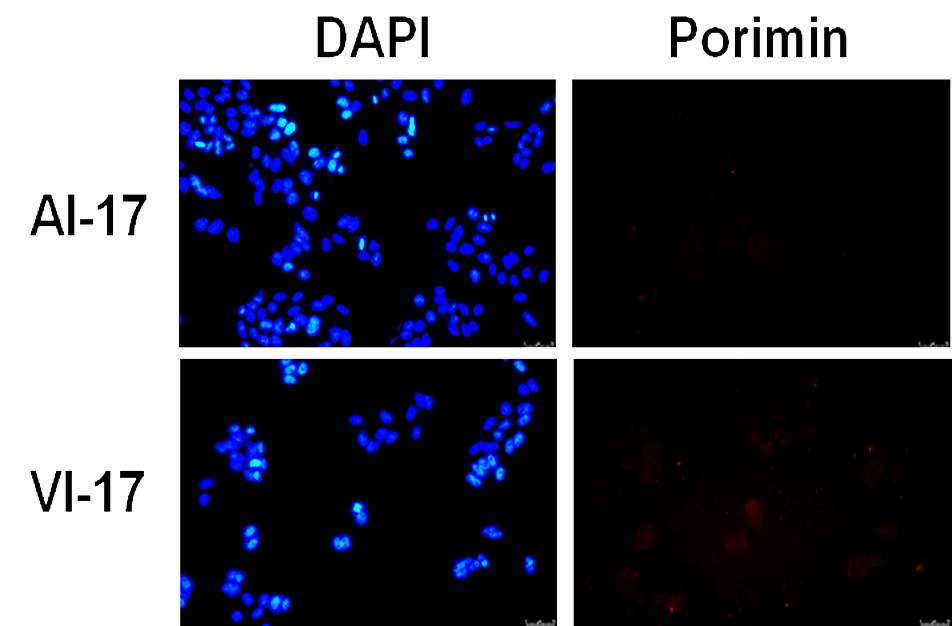
**

**Supplementary Figure 7G** The internalization process of VI-17 into the cytoplasm in Hep3B cells was observed as previous[^1^](#_ENREF_1). Briefly, Hep3B cells were cultured in glass- bottomed culture dishes (MatTek). During imaging, cells were cultured at 37°C in CO_2_ -independent medium (Invitrogen) containing 10% FBS and 25 μM FITC-VI-17 and were observed with the DeltaVision RT system (Applied Precision). Origin magnification: ×600 (yellow arrows indicated the peptide VI-17).

**
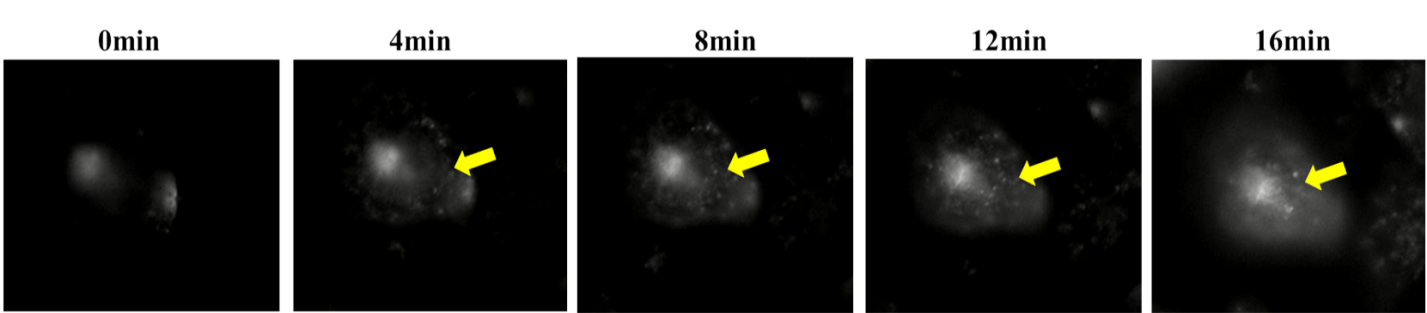
**

**1. Dou Z*, et al.* Dynamic localization of Mps1 kinase to kinetochores is essential for accurate spindle microtubule attachment. *Proceedings of the National Academy of Sciences of the United States of America* 112:E4546-4555 (2015).**
